# Effect of empagliflozin monotherapy on postprandial glucose and 24-hour glucose variability in Japanese patients with type 2 diabetes mellitus: a randomized, double-blind, placebo-controlled, 4-week study

**DOI:** 10.1186/s12933-014-0169-9

**Published:** 2015-01-30

**Authors:** Rimei Nishimura, Yuko Tanaka, Kazuki Koiwai, Kohei Inoue, Thomas Hach, Afshin Salsali, Søren S Lund, Uli C Broedl

**Affiliations:** Jikei University School of Medicine, Tokyo, Japan; Nippon Boehringer Ingelheim Co. Ltd, Osaki 2-1-1, ThinkPark Tower, Tokyo, 141-6017 Japan; EPS Corporation, Tokyo, Japan; Boehringer Ingelheim Pharma GmbH & Co. KG, Ingelheim, Germany; Boehringer Ingelheim Pharmaceuticals, Inc, Ridgefield, CT USA

**Keywords:** SGLT2 inhibitor, Continuous glucose monitoring, CGM

## Abstract

**Background:**

This study evaluated the effect of empagliflozin on postprandial glucose (PPG) and 24-hour glucose variability in Japanese patients with type 2 diabetes mellitus (T2DM).

**Methods:**

Patients (N = 60; baseline mean [SD] HbA1c 7.91 [0.80]%; body mass index 24.3 [3.2] kg/m^2^) were randomized to receive empagliflozin 10 mg (n = 20), empagliflozin 25 mg (n = 19) or placebo (n = 21) once daily as monotherapy for 28 days. A meal tolerance test and continuous glucose monitoring (CGM) for 24 hours were performed at baseline and on days 1 and 28. The primary endpoint was change from baseline in area under the glucose concentration-time curve 3 hours after breakfast (AUC_1–4h_ for PPG) at day 28.

**Results:**

Adjusted mean (95%) differences versus placebo in changes from baseline in AUC_1-4h_ for PPG at day 1 were −97.1 (−126.5, −67.8) mg · h/dl with empagliflozin 10 mg and −91.6 (−120.4, −62.8) mg · h/dl with empagliflozin 25 mg (both p < 0.001 versus placebo) and at day 28 were −85.5 (−126.0, −45.0) mg · h/dl with empagliflozin 10 mg and −104.9 (−144.8, −65.0) mg · h/dl with empagliflozin 25 mg (both p < 0.001 versus placebo). Adjusted mean (95% CI) differences versus placebo in change from baseline in 24-hour mean glucose (CGM) at day 1 were −20.8 (−27.0, −14.7) mg/dl with empagliflozin 10 mg and −23.9 (−30.0, −17.9) mg/dl with empagliflozin 25 mg (both p < 0.001 versus placebo) and at day 28 were −24.5 (−35.4, −13.6) mg/dl with empagliflozin 10 mg and −31.7 (−42.5,-20.9) mg/dl with empagliflozin 25 mg (both p < 0.001 versus placebo). Changes from baseline in mean amplitude of glucose excursions (MAGE; CGM) were not significantly different with either empagliflozin dose versus placebo at either timepoint. Curves of mean glucose (CGM) did not change between baseline and day 1 or 28 with placebo, but shifted downward with empagliflozin. Percentage of time with glucose ≥70 to <180 mg/dl increased from 52.0% at baseline to 77.0% at day 28 with empagliflozin 10 mg and from 55.0% to 81.1% with empagliflozin 25 mg, without increasing time spent with hypoglycemia.

**Conclusion:**

Empagliflozin for 28 days reduced PPG from the first day and improved daily blood glucose control in Japanese patients with T2DM.

**Trial registration:**

Clinicaltrials.gov NCT01947855

**Electronic supplementary material:**

The online version of this article (doi:10.1186/s12933-014-0169-9) contains supplementary material, which is available to authorized users.

## Background

The prevalence of diabetes in Japan is increasing [1]. Cardiovascular and all-cause mortality are increased in Japanese patients with diabetes [2].

Postprandial hyperglycemia is common in patients with type 2 diabetes (T2DM) [3,4]. Control of postprandial glucose (PPG) helps patients to achieve HbA1c goals [5,6], and some guidelines for the management of T2DM provide specific targets for PPG [7-9]. Postprandial hyperglycemia is an independent risk factor for cardiovascular disease [10,11], possibly due to the oxidative stress, endothelial dysfunction and overexpression of adhesion molecules triggered by acute hyperglycemia and glucose fluctuations [12,13]. Daily glucose fluctuations may also increase the risk of microvascular and macrovascular complications associated with T2DM [14,15] while severe hypoglycemia is associated with increased mortality [16,17].

Inhibition of the sodium glucose cotransporter 2 (SGLT2), located in the proximal tubule, reduces renal glucose reabsorption, leading to increased urinary glucose excretion and reduced hyperglycemia in patients with T2DM [18,19]. Empagliflozin is a selective and potent SGLT2 inhibitor [20]. In international Phase III trials in patients with T2DM, 24 weeks’ treatment with empagliflozin given as monotherapy or as add-on therapy for 24 weeks was well tolerated and significantly reduced glycated hemoglobin (HbA1c), fasting plasma glucose (FPG), body weight and systolic blood pressure (SBP) versus placebo [21-24]. In Japanese patients with T2DM, empagliflozin monotherapy for 52 weeks led to sustained reductions in HbA1c, FPG, body weight and SBP [25,26]. The effect of empagliflozin on 24-hour glycemic variability in patients with T2DM has not been assessed.

This study was conducted to evaluate the effect of empagliflozin 10 mg and 25 mg once daily as monotherapy for 28 days on PPG and 24-hour glycemic variability in Japanese patients with T2DM.

## Methods

This was a randomized, double-blind, placebo-controlled, parallel-group study conducted at two sites in Japan. The clinical trial protocol was approved by the Institutional Review Boards of the participating centers, and complied with the Declaration of Helsinki in accordance with the International Conference on Harmonisation Harmonised Tripartite Guideline for Good Clinical Practice. All patients provided written informed consent. The trial was registered with ClinicalTrials.gov (NCT01947855).

### Patients

Japanese patients with T2DM aged ≥20 and ≤74 years, with a body mass index (BMI) ≤40 kg/m^2^, who were on a diet and exercise regimen and were drug-naïve (no anti-diabetes agents for ≥12 weeks prior to consent) or treated with 1 oral anti-diabetes agent (except a sulfonylurea at > half maximum approved daily dose, or a thiazolidinedione) at an unchanged dose for ≥12 weeks prior to consent, were eligible for inclusion. At screening, drug-naïve patients were required to have HbA1c ≥7% and ≤10% and patients treated with 1 oral anti-diabetes agent were required to have HbA1c ≥7% and ≤9.5%. All patients were required to have HbA1c ≥7% to ≤10% at the start of the placebo run-in period.

Exclusion criteria included uncontrolled hyperglycemia (glucose level >240 mg/dl) after an overnight fast confirmed by a second measurement; acute coronary syndrome, stroke or transient ischemic attack ≤20 weeks prior to randomization; indication of liver disease (alanine aminotransferase, alkaline aminotransferase or alkaline phosphatase levels >3 times the upper limit of normal during screening, washout or run-in); impaired renal function (estimated glomerular filtration rate [eGFR] <60 ml/min/1.73 m^2^ according to Japanese estimation equation [27]) during screening, washout or run-in; gastrointestinal surgeries that induce chronic malabsorption; treatment with insulin, glucagon-like peptide-1 (GLP-1) analogues, sulfonylurea at > half the daily maximum approved dose or thiazolidinedione within 12 weeks prior to consent; treatment with anti-obesity drugs within 12 weeks prior to consent; use of any treatment at screening leading to unstable body weight; treatment with systemic steroids at time of consent; change in dosage of thyroid hormones within 6 weeks prior to consent; alcohol or drug abuse within 12 weeks of consent; investigational drug intake in another trial within 30 days of consent.

### Randomization and interventions

**Figure 1:**
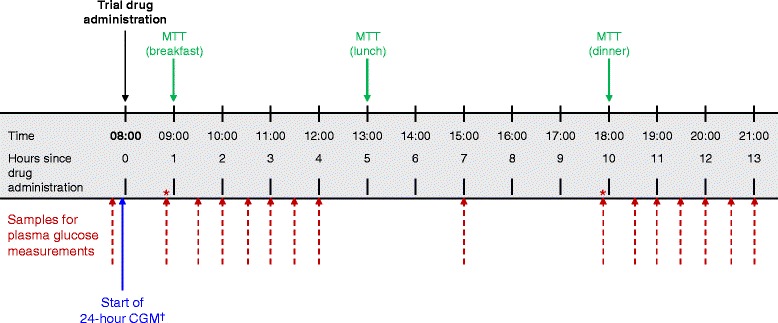
**MTT and plasma glucose sampling schedule at baseline, day 1 and day 28.** *Shortly before MTT; ^†^CGM was started shortly before trial drug administration and continued until 24 hours after trial drug administration. MTT: meal tolerance test. CGM, continuous glucose monitoring.

All patients underwent a 2-week, open-label, placebo run-in period. Patients pre-treated with an oral anti-diabetes agent underwent a 2-week washout period prior to the placebo run-in. Following the run-in period, eligible patients were randomized (in a 1:1:1 ratio) to receive empagliflozin 10 mg, empagliflozin 25 mg, or placebo for 28 days. Patients were monitored at the trial site from days −2 to 2 and days 27 to 29. Blinded 24-hour continuous glucose monitoring (CGM) and a meal tolerance test (MTT) were performed at day −1, day 1 (of treatment) and day 28 (Figure 1). Patients were assigned to test meals providing 1440, 1600, or 1840 kcal/day, based on patient’s standard weight (Additional file 1: Table S1). Test meals contained 50–60% carbohydrate, 15–21% protein, and 21–35% fat (Additional file 1: Table S1). Plasma glucose profiles were determined at the timepoints shown in Figure 1.

### Endpoints

The primary endpoint was the change from baseline (day −1) in the area under the glucose concentration-time curve 3 hours after breakfast (AUC_1–4h_ for PPG) at day 28. Other efficacy endpoints were change from baseline in AUC_1–4h_ for PPG at day 1, change from baseline in AUC of glucose 3 hours after dinner (AUC_10-13h_ for PPG) at day 1 and day 28, change from baseline in 2-hour PPG after each meal (breakfast, lunch, dinner) at day 1 and day 28, change from baseline in FPG at day 2 and 29 and change from baseline in AUC_1-4h_ and AUC_10-13h_ for postprandial insulin at day 1 and day 28. Endpoints measured from CGM at day 1 and day 28 were changes from baseline in 24-hour mean glucose, mean amplitude of glucose excursions (MAGE) [28] and the percentage of time with glucose ≥180 mg/dl, ≥70 to <180 mg/dl and <70 mg/dl per day. MAGE was calculated as the arithmetic mean difference between consecutive blood glucose peaks (between meals) and nadirs (between the peaks) when differences were >1 standard deviation of the mean glucose value in the same 24-hour period. Change from baseline in HbA1c was measured at day 29. Change from baseline in urinary excretion of 8-iso-prostaglandin F2α (8-iso-PGF2α; a marker of oxidative stress) in the fasting state and in the 24 hours after study drug administration was measured at day 28. Safety endpoints included changes in vital signs, weight, and clinical laboratory parameters, and adverse events (AEs; preferred terms coded according to the Medical Dictionary for Drug Regulatory Activities [MedDRA] version 16.1). AEs included all events with an onset after the first dose and up to 7 days after the last dose of study medication. Confirmed hypoglycemic AEs were defined as AEs with plasma glucose ≤70 mg/dL and/or requiring assistance. Events consistent with urinary tract infection (UTI), genital infection, and volume depletion were identified using prospectively defined search categories using 77, 89 and 8 preferred terms, respectively.

### Statistical analysis

Efficacy analyses were performed on the full analysis set (FAS) which included patients treated with ≥1 dose of study drug who had a baseline value for AUC_1-4h_ for PPG. Safety was assessed in the treated set (patients treated with ≥1 dose of study drug).

The primary endpoint was analyzed using an analysis of covariance (ANCOVA) model, with treatment, baseline eGFR and number of previous anti-diabetes medications as fixed effects and baseline HbA1c and baseline AUC_1-4h_ for PPG as linear covariates. Missing data were not imputed. In the hierarchical testing procedure, the superiority of empagliflozin 25 mg versus placebo was to be tested first, followed by empagliflozin 10 mg versus placebo if the first test was significant. Other efficacy endpoints were analyzed using the ANCOVA model described for the primary endpoint, with the baseline value for the endpoint in question as an additional linear covariate.

Safety analyses were descriptive, except for changes in lipid parameters, free fatty acids and blood ketone bodies, which were analyzed using ANCOVA.

Postprandial insulin data and triglyceride data were log-transformed prior to analysis.

A sample size of 20 patients per treatment group was required to provide power of 95% for the pair-wise comparison and an overall power of ≥90% to detect a 150 h · mg/dl treatment difference in AUC_1-4h_ for PPG for each empagliflozin dose compared to placebo, assuming a standard deviation of 120 h · mg/dl and a dropout rate of 2 patients per group.

## Results

### Patients

Of 78 patients screened, 60 patients were randomized and treated and comprised the FAS. One patient in the placebo group discontinued prematurely. Baseline characteristics were balanced across treatment groups (Table 1).

**Table 1:** **Patient demographics and baseline characteristics (full analysis set)**

	**Placebo**	**Empagliflozin 10 mg**	**Empagliflozin 25 mg**
N	21	20	19
Male	17 (81.0)	14 (70.0)	16 (84.2)
Age (years)	60.7 (10.8)	64.8 (5.9)	62.6 (7.8)
Time since diagnosis of type 2 diabetes			
≤5 years	9 (42.9)	4 (20.0)	5 (26.3)
>5 to 10 years	9 (42.9)	8 (40.0)	7 (36.8)
>10 years	3 (14.3)	8 (40.0)	7 (36.8)
Number of anti-diabetes medications			
0	18 (85.7)	16 (80.0)	17 (89.5)
1	3 (14.3)	4 (20.0)	2 (10.5)
Body weight (kg)	67.7 (10.0)	63.5 (10.6)	65.9 (12.1)
Body mass index (kg/m^2^)	24.9 (2.8)	24.1 (3.7)	24.0 (3.2)
HbA1c (%)	8.00 (0.82)	7.99 (0.83)	7.73 (0.75)
Fasting plasma glucose (mg/dl)*	154.5 (19.8)	151.0 (21.6)	151.9 (23.3)
AUC_1-4 h_ for postprandial glucose (mg · h/dl)	682.8 (91.2)	680.4 (92.2)	658.1 (116.2)
24-hour mean glucose (mg/dl)	184.1 (30.5)	181.3 (25.9)	178.4 (33.4)
Mean amplitude of glucose excursions, MAGE (mg/dl)	91.4 (26.4)	94.1 (18.5)	89.1 (29.7)
Estimated glomerular filtration rate (ml/min/1.73 m^2^) (Japanese estimation equation [27])	82.6 (12.8)	76.5 (11.1)	80.7 (9.3)
Systolic blood pressure (mmHg)*	119.8 (11.5)	119.1 (15.9)	124.0 (11.6)
Diastolic blood pressure (mmHg)*	71.8 (7.7)	70.7 (10.7)	74.7 (8.0)

Data are n (%) or mean (standard deviation). *Baseline for these parameters is day 1.

### Efficacy

**Figure 2:**
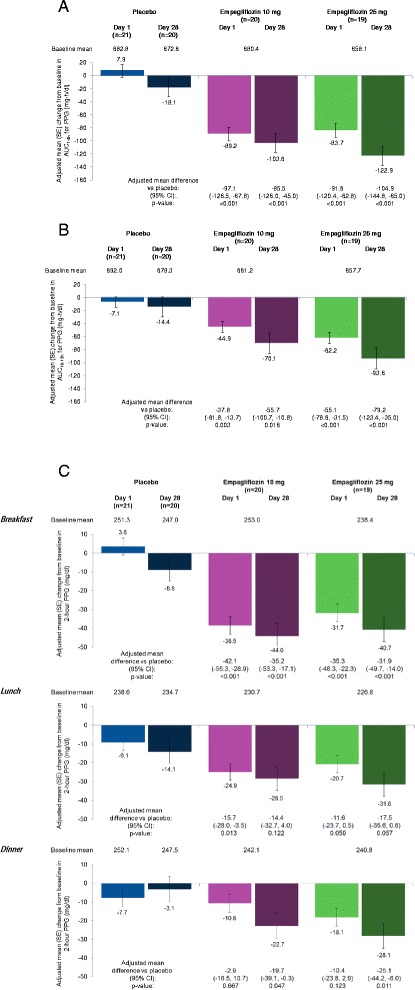
**Changes from baseline in (A) AUC**_**1-4 h**_ **for PPG, (B) AUC**_**10-13 h**_ **for PPG and (C) 2-hour PPG after each meal, based on analyses of covariance in the full analysis set.** CI, confidence interval; PPG, postprandial glucose; SE, standard error.

Compared with placebo, empagliflozin 10 mg and 25 mg led to significant reductions from baseline in AUC_1-4h_ for PPG at day 1 and at day 28 (Figure 2A) and in AUC_10-13h_ for PPG at day 1 and at day 28 (Figure 2B). Reductions in AUC_1-4h_ and AUC_10–13h_ for PPG at day 28 compared with placebo were greater with empagliflozin 25 mg than empagliflozin 10 mg (No statistical tests were performed on the differences between the empagliflozin 10 mg and 25 mg groups). Empagliflozin 10 mg and 25 mg reduced AUC_1–4h_ and AUC_10–13h_ for post-prandial insulin at day 1 and day 28, but changes in AUC_1–4h_ with empagliflozin 10 mg at day 28 were not significantly different to placebo (Table 2).

**Table 2:** **Changes in postprandial insulin after breakfast and dinner at day 1 and day 28**

	**Placebo (n = 20)**	**Empagliflozin 10 mg (n = 20)**	**Empagliflozin 25 mg (n = 19)**
**AUC**_**1–4h**_ **for postprandial insulin, μIU · h/ml**			
Baseline	66.4 (63.5)*	58.9 (55.6)	65.3 (46.2)
Relative change from baseline at day 1	1.2 (1.1, 1.3)	1.0 (0.9, 1.1)	1.0 (0.9, 1.0)
Difference vs placebo (95% CI)		0.8 (0.7, 0.9)	0.8 (0.7, 0.9)
p-value		<0.001	<0.001
Relative change from baseline at day 28	1.0 (0.9, 1.1)	0.9 (0.8, 0.9)	0.8 (0.7, 0.9)
Difference vs placebo (95% CI)		0.9 (0.8, 1.0)	0.8 (0.7, 0.9)
p-value		0.074	0.002
**AUC**_**10–13h**_ **for postprandial insulin, μIU · h/ml**			
Baseline	73.8 (56.6)†	60.7 (60.5)	70.0 (53.7)
Relative change from baseline at day 1	1.1 (1.0, 1.2)	0.9 (0.8, 1.0)	0.9 (0.8, 0.9)
Difference vs placebo (95% CI)		0.8 (0.8, 0.9)	0.8 (0.7, 0.9)
p-value		0.002	<0.001
Relative change from baseline at day 28	1.0 (0.9, 1.0)	0.8 (0.8, 0.9)	0.8 (0.7, 0.9)
Difference vs placebo (95% CI)		0.9 (0.8, 1.0)	0.8 (0.7, 0.9)
p-value		0.011	0.001

Log-transformed data. Baseline data are gMean (% gCV), change from baseline data are adjusted gMean ratio (95% CI) based on analysis of covariance (ANCOVA) in the full analysis set. *63.8 (65.5) for day 1 analysis (n = 21). ^†^72.8 (55.5) for day 1 analysis (n = 21).

Changes from baseline in 2-hour PPG were significantly greater with empagliflozin 10 mg and 25 mg compared with placebo after breakfast at day 1 and day 28 (Figure 2C). Changes from baseline in 2-hour PPG after lunch were significantly greater with empagliflozin 10 mg compared with placebo at day 1, but did not reach significance versus placebo with empagliflozin 10 mg at day 28 or with empagliflozin 25 mg at day 1 or day 28 (Figure 2C). Changes from baseline in 2-hour PPG after dinner were significantly different with empagliflozin 10 mg and empagliflozin 25 mg compared with placebo at day 28 but not at day 1 (Figure 2C).

**Figure 3:**
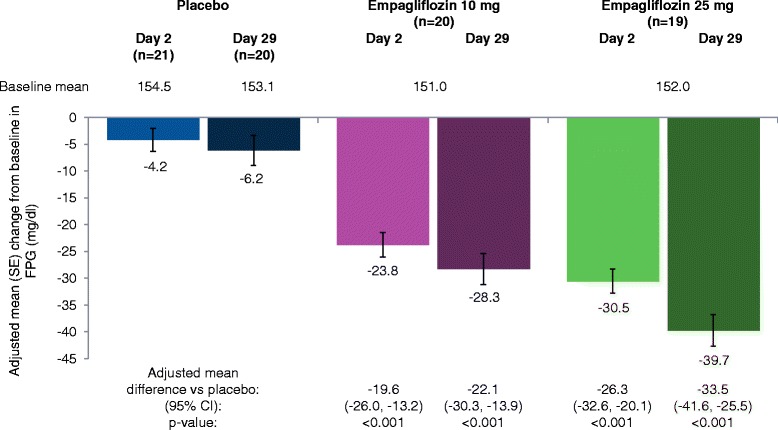
**Change from baseline in FPG at day 2 and day 29 based on analyses of covariance in the full analysis set.** CI, confidence interval; FPG, fasting plasma glucose; SE, standard error.

Empagliflozin 10 mg and 25 mg led to significant reductions from baseline in FPG compared with placebo at day 2 and at day 29 (Figure 3). Reductions from baseline in FPG at day 29 compared with placebo were greater with empagliflozin 25 mg than empagliflozin 10 mg.

**Figure 4:**
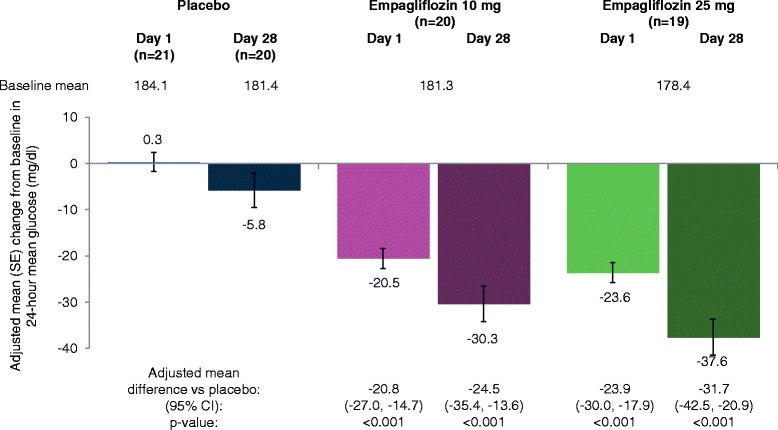
**Change from baseline in 24-hour mean glucose by CGM based on analyses of covariance in the full analysis set.** CGM, continuous glucose monitoring; CI, confidence interval; SE, standard error.

**Figure 5:**
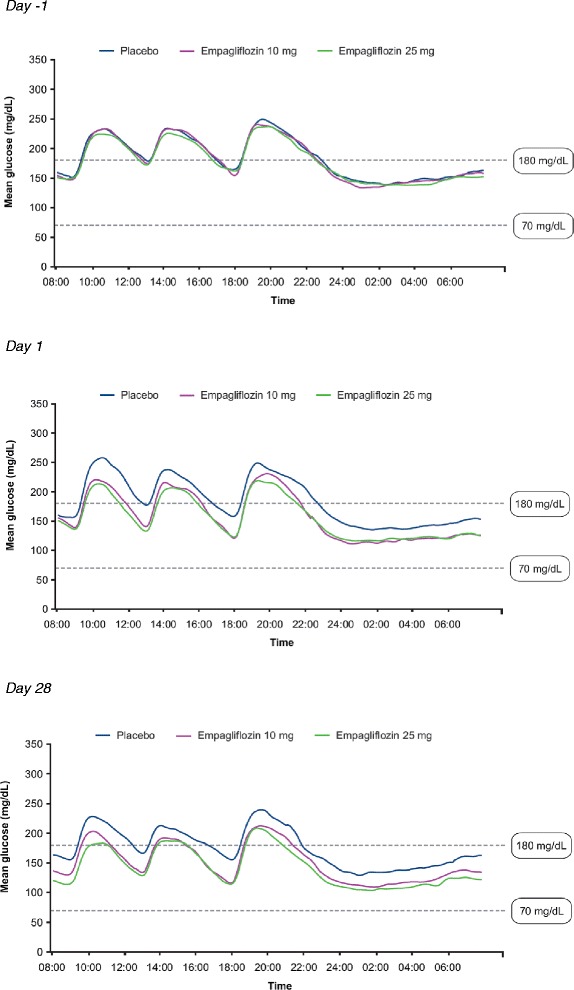
**Mean glucose over 24 hours by CGM.** CGM, continuous glucose monitoring.

**Figure 6:**
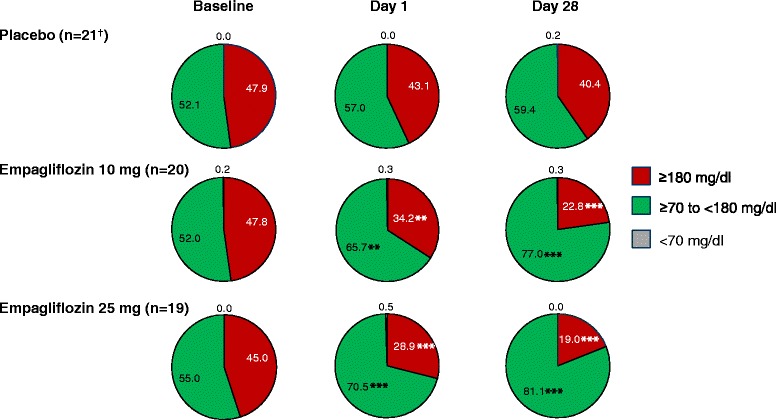
**Percentage of time with glucose level ≥180 mg/dl, ≥70 to <180 mg/dl, and <70 mg/dl, based on analyses of covariance in the full analysis set.** Baseline data are means, day 1 and day 28 data are adjusted means. **p<0.01; ***p<0.001 for difference vs placebo in change from baseline; ^†^n=20 at day 28.

Empagliflozin 10 mg and 25 mg led to significant reductions from baseline in 24-hour mean glucose compared with placebo at day 1 and at day 28 (Figure 4). Reductions from baseline in 24-hour mean glucose compared with placebo at day 28 were greater with empagliflozin 25 mg than empagliflozin 10 mg. Mean glucose levels over 24 hours by CGM at baseline, day 1 and day 28 are shown in Figure 5. A reduction from baseline (downward shift) in mean glucose levels at all timepoints over 24 hours was evident from day 1 with empagliflozin, and reductions from baseline seemed to be slightly greater with empagliflozin 25 mg than empagliflozin 10 mg (Figure 5). At day 1, adjusted mean (SE) changes from baseline in MAGE were 15.1 (3.5), 11.0 (3.7) and 8.9 (3.7) mg/dl with placebo, empagliflozin 10 mg and empagliflozin 25 mg, respectively. At day 28, adjusted mean (SE) changes from baseline in MAGE were −4.7 (4.5), −3.7 (4.6) and −2.2 (4.7) mg/dl with placebo, empagliflozin 10 mg and empagliflozin 25 mg, respectively. Differences were not statistically significant with either empagliflozin dose compared with placebo at either timepoint. Compared with placebo, empagliflozin 10 mg and 25 mg reduced the percentage of time with glucose ≥180 mg/dl (p < 0.01), and increased the percentage of time with normoglycemia (glucose ≥70 to <180 mg/dl) (p < 0.01) without significantly increasing the percentage of time with hypoglycemia (glucose <70 mg/dl) (Figure 6; Additional file 1: Table S2).

At day 29, adjusted mean (SE) changes from baseline in HbA1c were −0.11 (0.06)% with placebo compared with −0.46 (0.06)% with empagliflozin 10 mg (adjusted mean [95% CI] difference: −0.35% [−0.52, −0.19]; p < 0.001) and −0.63 (0.06)% with empagliflozin 25 mg (adjusted mean [95% CI] difference: −0.52% [−0.68, −0.35]; p < 0.001).

Consistent with reductions in PPG, the excretion of 8-iso-PGF2α, a marker of oxidative stress, was significantly reduced from baseline with empagliflozin 10 mg and 25 mg compared with placebo at day 28 in the fasting state (Table 3). Reductions from baseline in the excretion of 8-iso-PGF2α in the 24 hours after study drug administration were only significantly different with empagliflozin 25 mg compared with placebo at day 28 (Table 3).

**Table 3:** **Changes in urinary excretion of 8-iso-PGF2α at day 28**

	**Placebo (n = 20)**	**Empagliflozin 10 mg (n = 20)**	**Empagliflozin 25 mg (n = 19)**
**Fasting state, pg/ml**			
Baseline	197.8 (27.0)	194.6 (29.4)	146.5 (18.5)
Change from baseline at day 28	40.6 (22.6)	−48.1 (23.3)	−33.5 (23.7)
Difference vs placebo (95% CI)		−88.6 (−154.6, −22.7)	−74.0 (−139.8, −8.2)
p-value		0.010	0.028
**In 24 hours after drug administration, pg/ml**			
Baseline	115.5 (11.0)	138.3 (20.6)	148.6 (21.7)
Change from baseline at day 28	−3.7 (10.3)	−28.4 (10.4)	−46.8 (10.6)
Difference vs placebo (95% CI)		−24.7 (−54.5, 5.2)	−43.1 (−72.9, −13.3)
p-value		0.103	0.006

Baseline data are mean (standard error [SE]), change from baseline data are adjusted mean (SE) based on analysis of covariance (ANCOVA) in the full analysis set.

### Safety

AEs were reported in 2 patients (9.5%) on placebo, 3 patients (15.0%) on empagliflozin 10 mg and 3 patients (15.8%) on empagliflozin 25 mg. No severe AEs, serious AEs, or AEs leading to discontinuation occurred. No hypoglycemic AEs were reported. One patient (on empagliflozin 25 mg) experienced an AE consistent with genital infection (bartholinitis). No AEs consistent with UTI or volume depletion were reported. No AEs of diabetic ketoacidosis or those related to abnormal ketone body levels were reported.

At day 29, weight was reduced from baseline by 0.9 kg, 1.7 kg and 2.1 kg with placebo, empagliflozin 10 mg and 25 mg, respectively (Additional file 1: Table S3). Acute changes in SBP and diastolic BP (DBP) (at day 2) with empagliflozin compared with placebo were small, and more pronounced reductions were observed at day 29 (Additional file 1: Table S3). In contrast, pulse rate appeared to increase with empagliflozin compared with placebo at day 2, but changes from baseline in pulse rate were similar between empagliflozin and placebo at day 29 (Additional file 1: Table S3).

Compared with placebo, there were no significant differences in changes from baseline in total cholesterol or LDL-cholesterol with empagliflozin 10 mg or 25 mg (Table 4). Compared with placebo, HDL-cholesterol was significantly increased with empagliflozin 10 mg and 25 mg, and triglycerides were significantly reduced with empagliflozin 10 mg and 25 mg, at day 29. There were significant increases from baseline in free fatty acids with empagliflozin 25 mg, but not with empagliflozin 10 mg, compared with placebo at day 29. There were significant increases from baseline in blood ketone bodies with empagliflozin 10 mg and 25 mg compared with placebo at day 29 (Table 4).

**Table 4:** **Changes in fasting serum lipids and ketone bodies at day 29**

	**Placebo (n = 20)**	**Empagliflozin 10 mg (n = 20)**	**Empagliflozin 25 mg (n = 19)**
**Total cholesterol, mg/dl**			
Baseline	212.8 (7.7)	200.1 (8.4)	208.7 (9.1)
Change from baseline at day 29	−0.8 (3.6)	3.2 (3.7)	1.1 (3.7)
Difference vs placebo (95% CI)		4.0 (−6.6, 14.6)	1.9 (−8.6, 12.3)
p-value		0.455	0.722
**HDL-cholesterol, mg/dl**			
Baseline	46.8 (2.2)	47.6 (2.8)	46.8 (2.8)
Change from baseline at day 29	0.1 (1.2)	4.7 (1.3)	7.4 (1.3)
Difference vs placebo (95% CI)		4.5 (1.0, 8.1)	7.2 (3.7, 10.8)
p-value		0.014	<0.001
**LDL-cholesterol, mg/dl**			
Baseline	128.7 (6.3)	124.9 (8.4)	130.5 (8.5)
Change from baseline at day 29	−1.3 (3.2)	3.4 (3.3)	4.3 (3.3)
Difference vs placebo (95% CI)		4.7 (−4.6, 14.0)	5.7 (−3.5, 14.8)
p-value		0.314	0.221
Triglycerides, mg/dl*			
Baseline	159.6 (51.0)	131.5 (34.9)	142.2 (48.0)
Relative change from baseline at day 29	1.0 (0.9, 1.1)	0.8 (0.7, 0.9)	0.7 (0.6, 0.8)
Difference vs placebo (95% CI)		0.8 (0.7, 1.0)	0.7 (0.6, 0.8)
p-value		0.037	<0.001
**Free fatty acids, mg/dl**			
Baseline	10.8 (0.8)	9.6 (0.7)	9.3 (0.6)
Change from baseline at day 29	3.1 (0.8)	4.8 (0.8)	7.4 (0.8)
Difference vs placebo (95% CI)		1.7 (−0.6, 4.1)	4.3 (2.0, 6.7)
p-value		0.149	<0.001
**Ketone bodies, μmol/l**			
Baseline	86.8 (10.2)	78.1 (12.0)	69.4 (10.8)
Change from baseline at day 29	−12.2 (44.0)	139.5 (44.7)	408.0 (45.5)
Difference vs placebo (95% CI)		151.7 (24.4, 279.0)	420.2 (293.3, 547.2)
p-value		0.021	<0.001

Unless otherwise indicated, baseline data are mean (standard error [SE]), change from baseline data are adjusted mean (SE) based on analysis of covariance (ANCOVA) in the treated set. Fasting measurements. *Log-transformed data; baseline data are gMean (%CV) and change from baseline data are adjusted gMean ratio (95% CI).

No clinically relevant changes in electrolytes (sodium, potassium, calcium, magnesium, phosphate) were observed in any group at the end of treatment (Additional file 1: Table S4). Changes from baseline in hematocrit and eGFR were generally small in all groups (Additional file 1: Table S4).

## Conclusions

This study was conducted to evaluate the effect of empagliflozin as monotherapy for 28 days on PPG and 24-hour glycemic variability in Japanese patients with T2DM. Significant reductions from baseline in AUC_1-4h_ for PPG were observed after acute and subchronic treatment with empagliflozin, with 80–90% of the reduction in AUC_1-4h_ for PPG already achieved at day 1.

At day 28, although the reductions from baseline in AUC for PPG with empagliflozin observed after dinner were of a lower magnitude than those observed after breakfast, the reductions observed after dinner were significant. These observations were consistent with reductions in 2-hour PPG. The sustained effect of empagliflozin on PPG from morning to evening support once-daily administration of empagliflozin.

Of note, the reduction in PPG in this study was accompanied by a reduction in postprandial insulin levels. In contrast to insulin secretagogues and incretins, empagliflozin’s mode of action is independent of beta-cell function and insulin secretion [18]. By increasing urinary glucose excretion, empagliflozin reduces plasma glucose levels leading to a reduction in plasma insulin levels [29].

CGM can provide valuable information on the magnitude and duration of glucose fluctuations [30]. In this study, empagliflozin improved daily blood glucose control measured using CGM, with the curves of mean 24-hour glucose lower at day 1 and day 28 than at baseline. Consistent with changes in FPG, PPG and HbA1c, slightly greater reductions in 24-hour mean glucose and mean glucose levels over 24 hours were observed with empagliflozin 25 mg compared with empagliflozin 10 mg at day 28. Empagliflozin had a significant effect on FPG as well as PPG, and the reductions in PPG were not substantially different to the reductions in FPG. Therefore, the curves of mean 24-hour glucose with empagliflozin showed a parallel shift downward and MAGE was not significantly reduced by empagliflozin. Variable responses in FPG and PPG have been observed with empagliflozin in other clinical trials in patients with T2DM [21,22]. Further CGM data with SGLT2 inhibitors in patients with T2DM are needed to illuminate the effect of this class of drugs on MAGE.

Tight glucose control is important to reduce the risk of micro- and macrovascular complications [9], and to avoid the adverse effects on morbidity, mortality and quality of life associated with hypoglycemia [31]. Importantly, CGM measurements in this study showed that empagliflozin increased the time patients spent with normoglycemia without increasing the time spent at a hypoglycemic level.

Treatment with empagliflozin is consistently associated with weight loss in patients with T2DM [21-26]. This reflects loss of both trunk fat and limb fat, and reductions in both abdominal visceral and subcutaneous adipose tissue [32]. After 2 years’ treatment with empagliflozin 25 mg as add-on to metformin, approximately 90% of the weight loss observed was due to fat loss [32]. Empagliflozin-induced urinary glucose excretion results in calorie loss and reduced plasma glucose levels with an increased glucagon-to-insulin ratio [29], leading to lipolysis, increased free fatty acid levels and ketogenesis. The most common causes of ketosis are physiological conditions, in which mild to moderate elevations of circulating ketone bodies occur in response to fasting or prolonged exercise, with ketone body levels not uncommonly rising to the range of 1 ± 2 mM [33,34]. In this study, the average increase in ketone bodies was modest, with adjusted mean levels of 218, 486 and 66 μmol/l for empagliflozin 10 mg, empagliflozin 25 mg and placebo, respectively, at day 29. The highest level of ketone bodies observed in our study in an individual patient was 1449 μmol/l, which is comparable with levels of up to about 1300 μmol/l reported for subjects without diabetes after an overnight fast [35]. Diabetic ketoacidosis is typically accompanied by levels of ketone bodies >3000 μmol/l [33] and develops almost exclusively in states of absolute insulin deficiency. In contrast, the lowering of insulin levels with empagliflozin is probably secondary to the reduction in plasma glucose levels via increased urinary glucose excretion, which is accompanied by an improvement in beta-cell function [29]. Therefore, the empagliflozin-induced increase in ketone bodies most likely reflects an adaptive change, with ketone levels in the range of physiological conditions, which is unlikely to put patients at risk of ketoacidosis in the absence of absolute (endogenous or exogenous) insulin deficiency or extreme (ketogenic) diets.

Patients with T2DM have an increased risk of developing cardiovascular events compared with the general population [36], which is related to the prevalence of the classical cardiovascular risk factors of hypertension and dyslipidemia, in addition to other important factors such as glycemic control, oxidative stress, and obesity [37]. Elevated PPG is an independent risk factor for cardiovascular disease [10,11]; however, improvements in PPG have not been shown to translate into reduced risk of cardiovascular disease [38]. Empagliflozin improves glycemic control with a low risk of hypoglycemia, leads to weight loss and reduces blood pressure, possibly due to diuretic effects, weight loss, or direct vascular effects [21-26,39,40]; further, as demonstrated in this study, empagliflozin reduces PPG and 8-iso-PGF2α, a marker of oxidative stress that is an independent risk marker for cardiovascular disease [41]. A cardiovascular outcome trial (EMPA-REG OUTCOME™; NCT01131676) is investigating the effect of empagliflozin in patients with T2DM and high cardiovascular risk [42].

In conclusion, empagliflozin 10 mg or 25 mg as monotherapy for 28 days significantly reduced PPG and FPG and improved daily blood glucose control in Japanese patients with T2DM, without increasing time spent with a hypoglycemic blood glucose level.

## Additional file

Additional file 1: Table S1. Test meals. **Table S2.** Changes in percentage of time with glucose level ≥180 mg/dl, ≥70 to <180 mg/dl, and <70 mg/dl. **Table S3.** Changes in blood pressure, pulse rate and weight. **Table S4.** Laboratory measurements.

## Abbreviations

AE: Adverse event
ANCOVA: Analysis of covariance
AUC: Area under the glucose concentration-time curve
BMI: Body mass index
CGM: Continuous glucose monitoring
CI: Confidence interval
DBP: Diastolic blood pressure
eGFR: Estimated glomerular filtration rate
FAS: Full analysis set
FPG: Fasting plasma glucose
HbA1c: Glycated hemoglobin
HDL-cholesterol: High-density lipoprotein cholesterol
LDL-cholesterol: Low-density lipoprotein cholesterol
MAGE: Mean amplitude of glucose excursions
MedDRA: Medical Dictionary for Drug Regulatory Activities
MTT: Meal tolerance test
PPG: Postprandial glucose
SBP: Systolic blood pressure
SD: Standard deviation
SE: Standard error
SGLT2: Sodium glucose cotransporter 2
T2DM: Type 2 diabetes mellitus
UTI: Urinary tract infection

## References

[1] Guariguata L, Whiting DR, Hambleton I, Beagley J, Linnenkamp U, Shaw JE (2014). Global estimates of diabetes prevalence for 2013 and projections for 2035. Diabetes Res Clin Pract.

[2] Oba S, Nagata C, Nakamura K, Takatsuka N, Shimizu H (2008). Self-reported diabetes mellitus and risk of mortality from all causes, cardiovascular disease, and cancer in Takayama: a population-based prospective cohort study in Japan. J Epidemiol.

[3] Bonora E, Corrao G, Bagnardi V, Ceriello A, Comaschi M, Montanari P, Meigs JB (2006). Prevalence and correlates of post-prandial hyperglycaemia in a large sample of patients with type 2 diabetes mellitus. Diabetologia.

[4] Bonora E, Calcaterra F, Lombardi S, Bonfante N, Formentini G, Bonadonna RC, Muggeo M (2001). Plasma glucose levels throughout the day and HbA(1c) interrelationships in type 2 diabetes: implications for treatment and monitoring of metabolic control. Diabetes Care.

[5] Woerle HJ, Neumann C, Zschau S, Tenner S, Irsigler A, Schirra J, Gerich JE, Goke B (2007). Impact of fasting and postprandial glycemia on overall glycemic control in type 2 diabetes: importance of postprandial glycemia to achieve target HbA1c levels. Diabetes Res Clin Pract.

[6] Peter R, Dunseath G, Luzio SD, Owens DR (2013). Estimates of the relative and absolute diurnal contributions of fasting and post-prandial plasma glucose over a range of hyperglycaemia in type 2 diabetes. Diabetes Metab.

[7] Handelsman Y, Mechanick JI, Blonde L, Grunberger G, Bloomgarden ZT, Bray GA, Dagogo-Jack S, Davidson JA, Einhorn D, Ganda O (2011). American association of clinical endocrinologists medical guidelines for clinical practice for developing a diabetes mellitus comprehensive care plan. Endocr Pract.

[8] International Diabetes Federation: Guideline for management of postmeal glucose in diabetes [https://www.idf.org/2011-guideline-management-postmeal-glucose-diabetes] 10.1111/j.1464-5491.2008.02565.x PMC2701558 19046192

[9] Inzucchi SE, Bergenstal RM, Buse JB, Diamant M, Ferrannini E, Nauck M, Peters AL, Tsapas A, Wender R, Matthews DR (2012). Management of hyperglycemia in type 2 diabetes: a patient-centered approach. Position statement of the American Diabetes Association (ADA) and the European Association for the Study of Diabetes (EASD). Diabetes Care.

[10] Cavalot F, Petrelli A, Traversa M, Bonomo K, Fiora E, Conti M, Anfossi G, Costa G, Trovati M (2006). Postprandial blood glucose is a stronger predictor of cardiovascular events than fasting blood glucose in type 2 diabetes mellitus, particularly in women: lessons from the San Luigi Gonzaga diabetes study. J Clin Endocrinol Metab.

[11] Cavalot F, Pagliarino A, Valle M, Di ML, Bonomo K, Massucco P, Anfossi G, Trovati M (2011). Postprandial blood glucose predicts cardiovascular events and all-cause mortality in type 2 diabetes in a 14-year follow-up: lessons from the San Luigi Gonzaga diabetes study. Diabetes Care.

[12] Ceriello A, Esposito K, Piconi L, Ihnat MA, Thorpe JE, Testa R, Boemi M, Giugliano D (2008). Oscillating glucose is more deleterious to endothelial function and oxidative stress than mean glucose in normal and type 2 diabetic patients. Diabetes.

[13] Kawano H, Motoyama T, Hirashima O, Hirai N, Miyao Y, Sakamoto T, Kugiyama K, Ogawa H, Yasue H (1999). Hyperglycemia rapidly suppresses flow-mediated endothelium-dependent vasodilation of brachial artery. J Am Coll Cardiol.

[14] Sartore G, Chilelli NC, Burlina S, Lapolla A (2013). Association between glucose variability as assessed by continuous glucose monitoring (CGM) and diabetic retinopathy in type 1 and type 2 diabetes. Acta Diabetol.

[15] Su G, Mi S, Tao H, Li Z, Yang H, Zheng H, Zhou Y, Ma C (2011). Association of glycemic variability and the presence and severity of coronary artery disease in patients with type 2 diabetes. Cardiovasc Diabetol.

[16] Bonds DE, Miller ME, Bergenstal RM, Buse JB, Byington RP, Cutler JA, Dudl RJ, Ismail Beigi F, Kimel AR, Hoogwerf B (2010). The association between symptomatic, severe hypoglycaemia and mortality in type 2 diabetes: retrospective epidemiological analysis of the ACCORD study. BMJ.

[17] Hsu PF, Sung SH, Cheng HM, Yeh JS, Liu WL, Chan WL, Chen CH, Chou P, Chuang SY (2013). Association of clinical symptomatic hypoglycemia with cardiovascular events and total mortality in type 2 diabetes: a nationwide population-based study. Diabetes Care.

[18] DeFronzo RA, Davidson JA, del Prato S (2012). The role of the kidneys in glucose homeostasis: a new path towards normalizing glycaemia. Diabetes Obes Metab.

[19] Kanada S, Koiwai K, Taniguchi A, Sarashina A, Seman L, Woerle HJ (2013). Pharmacokinetics, pharmacodynamics, safety and tolerability of 4 weeks’ treatment with empagliflozin in Japanese patients with type 2 diabetes mellitus. J Diabetes Investig.

[20] Grempler R, Thomas L, Eckhardt M, Himmelsbach F, Sauer A, Sharp DE, Bakker RA, Mark M, Klein T, Eickelmann P (2012). Empagliflozin, a novel selective sodium glucose cotransporter-2 (SGLT-2) inhibitor: characterisation and comparison with other SGLT-2 inhibitors. Diabetes Obes Metab.

[21] Häring H-U, Merker L, Seewaldt-Becker E, Weimer M, Meinicke T, Woerle HJ, Broedl UC (2013). Empagliflozin as add-on to metformin plus sulfonylurea in patients with type 2 diabetes: a 24-week randomized, double-blind, placebo-controlled trial. Diabetes Care.

[22] Häring H-U, Merker L, Seewaldt-Becker E, Weimer M, Meinicke T, Broedl UC, Woerle HJ (2014). Empagliflozin as add-on to metformin in patients with type 2 diabetes: a 24-week, randomized, double-blind, placebo-controlled trial. Diabetes Care.

[23] Kovacs CS, Seshiah V, Swallow R, Jones R, Rattunde H, Woerle HJ, Broedl UC (2014). Empagliflozin improves glycaemic and weight control as add-on therapy to pioglitazone or pioglitazone plus metformin in patients with type 2 diabetes: a 24-week, randomized, placebo-controlled trial. Diabetes Obes Metab.

[24] Roden M, Weng J, Eilbracht J, Delafont B, Kim G, Woerle HJ, Broedl UC (2013). Empagliflozin monotherapy with sitagliptin as an active comparator in patients with type 2 diabetes: a randomised, double-blind, placebo-controlled, phase 3 trial. Lancet Diabetes Endocrinol.

[25] Kadowaki T, Haneda M, Inagaki N, Terauchi Y, Taniguchi A, Koiwai K, Rattunde H, Woerle HJ, Broedl UC (2014). Empagliflozin monotherapy in Japanese patients with type 2 diabetes mellitus: a randomized, 12-week, double-blind, placebo-controlled, phase II trial. Adv Ther.

[26] Woerle HJ, Kadowaki T, Haneda M, Inagaki A, Sakamoto M, Koiwai K, Rattunde H, Broedl UC (2013). Safety and efficacy of empagliflozin monotherapy in a 52-week study in Japanese patients with type 2 diabetes mellitus. Diabetologia.

[27] Matsuo S, Imai E, Horio M, Yasuda Y, Tomita K, Nitta K, Yamagata K, Tomino Y, Yokoyama H, Hishida A (2009). Revised equations for estimated GFR from serum creatinine in Japan. Am J Kidney Dis.

[28] Service FJ, Molnar GD, Rosevear JW, Ackerman E, Gatewood LC, Taylor WF (1970). Mean amplitude of glycemic excursions, a measure of diabetic instability. Diabetes.

[29] Ferrannini E, Muscelli E, Frascerra S, Baldi S, Mari A, Heise T, Broedl UC, Woerle HJ (2014). Metabolic response to sodium-glucose cotransporter 2 inhibition in type 2 diabetic patients. J Clin Invest.

[30] Klonoff DC (2005). Continuous glucose monitoring: roadmap for 21st century diabetes therapy. Diabetes Care.

[31] Barnett AH, Cradock S, Fisher M, Hall G, Hughes E, Middleton A (2010). Key considerations around the risks and consequences of hypoglycaemia in people with type 2 diabetes. Int J Clin Pract.

[32] Ridderstrale M, Andersen KR, Zeller C, Kim G, Woerle HJ, Broedl UC (2014). Comparison of empagliflozin and glimepiride as add-on to metformin in patients with type 2 diabetes: a 104-week randomised, active-controlled, double-blind, phase 3 trial. Lancet Diabetes Endocrinol.

[33] Laffel L (1999). Ketone bodies: a review of physiology, pathophysiology and application of monitoring to diabetes. Diabetes Metab Res Rev.

[34] Koeslag JH, Noakes TD, Sloan AW (1980). Post-exercise ketosis. J Physiol.

[35] Foster KJ, Alberti KG, Hinks L, Lloyd B, Postle A, Smythe P, Turnell DC, Walton R (1978). Blood intermediary metabolite and insulin concentrations after an overnight fast: reference ranges for adults, and interrelations. Clin Chem.

[36] Fox CS, Pencina MJ, Wilson PW, Paynter NP, Vasan RS, D’Agostino RB (2008). Lifetime risk of cardiovascular disease among individuals with and without diabetes stratified by obesity status in the Framingham heart study. Diabetes Care.

[37] Martin-Timon I, Sevillano-Collantes C, Segura-Galindo A, Del Canizo-Gomez FJ (2014). Type 2 diabetes and cardiovascular disease: have all risk factors the same strength?. World J Diabetes.

[38] Raz I, Wilson PW, Strojek K, Kowalska I, Bozikov V, Gitt AK, Jermendy G, Campaigne BN, Kerr L, Milicevic Z (2009). Effects of prandial versus fasting glycemia on cardiovascular outcomes in type 2 diabetes: the HEART2D trial. Diabetes Care.

[39] Baker WL, Smyth LR, Riche DM, Bourret EM, Chamberlin KW, White WB (2014). Effects of sodium-glucose co-transporter 2 inhibitors on blood pressure: a systematic review and meta-analysis. J Am Soc Hypertens.

[40] Tikkanen I, Narko K, Zeller C, Green A, Salsali A, Broedl UC, Woerle HJ: Empagliflozin reduces blood pressure in patients with type 2 diabetes and hypertension. *Diabetes Care* 2014 Epub ahead of print]. doi:10.2337/dc14-1096 10.2337/dc14-1096 25271206

[41] Schwedhelm E, Bartling A, Lenzen H, Tsikas D, Maas R, Brummer J, Gutzki FM, Berger J, Frolich JC, Boger RH (2004). Urinary 8-iso-prostaglandin F2alpha as a risk marker in patients with coronary heart disease: a matched case–control study. Circulation.

[42] Zinman B, Inzucchi SE, Lachin JM, Wanner C, Ferrari R, Fitchett D, Bluhmki E, Hantel S, Kempthorne Rawson J, Newman J (2014). Rationale, design, and baseline characteristics of a randomized, placebo-controlled cardiovascular outcome trial of empagliflozin (EMPA-REG OUTCOME). Cardiovasc Diabetol.

