# Supplementary material for: Effect of empagliflozin monotherapy on postprandial glucose and 24-hour glucose variability in Japanese patients with type 2 diabetes mellitus: a randomized, double-blind, placebo-controlled, 4-week study
Source: Cardiovasc Diabetol. 2015 Jan 30;14:11. doi: 10.1186/s12933-014-0169-9 (PMC4339254; doi:10.1186/s12933-014-0169-9)
Supplement: Additional file 1: Table S1. — Test meals. Table S2. Changes in percentage of time with glucose level ≥180 mg/dl, ≥70 to <180 mg/dl, and <70 mg/dl. Table S3. Changes in blood pressure, pulse rate and weight. Table S4. Laboratory measurements. [file 12933_2014_169_MOESM1_ESM.docx]

**Supplementary Table 1. Test meals**

|  | **1440 kcal/day** | **1600 kcal/day** | **1840 kcal/day** |
| --- | --- | --- | --- |
| **Breakfast** |  |  |  |
| Total calories (kcal) | 415 | 484 | 484 |
| Carbohydrate (% kcal) | 56.6 | 56.5 | 56.5 |
| Protein (% kcal) | 16.7 | 20.7 | 20.7 |
| Fat (% kcal) | 26.7 | 22.8 | 22.8 |
| **Lunch** |  |  |  |
| Total calories (kcal) | 490 | 511 | 697 |
| Carbohydrate (% kcal) | 56.7 | 58.2 | 53.9 |
| Protein (% kcal) | 15.8 | 15.4 | 16.8 |
| Fat (% kcal) | 27.5 | 26.4 | 29.3 |
| **Dinner** |  |  |  |
| Total calories (kcal) | 604 | 651 | 693 |
| Carbohydrate (% kcal) | 49.8 | 56.5 | 59.7 |
| Protein (% kcal) | 14.8 | 20.7 | 19.6 |
| Fat (% kcal) | 35.4 | 22.8 | 20.7 |

Patients were assigned to test meals according to the equation: kcal/day = standard body weight (22 x height [m] squared x 25 kcal/kg. Patients were assigned to the group closest to the calculated amount.

**Supplementary Table 2. Changes in percentage of time with glucose level ≥180 mg/dl, ≥70 to <180 mg/dl, and <70 mg/dl.**

|  | **Placebo  (n=20)** | **Empagliflozin 10 mg  (n=20)** | **Empagliflozin 25 mg**  **(n=19)** |
| --- | --- | --- | --- |
| **Percentage of time with glucose level ≥180 mg/dL per day** |  |  |  |
| Baseline | 45.7 (5.0)* | 47.8 (4.3) | 45.0 (6.2) |
| Change from baseline at day 1 | -3.8 (1.8) | -12.8 (1.9) | -18.0 (1.9) |
| Difference vs placebo (95% CI) |  | -8.9 (-14.3, -3.5) | -14.2 (-19.5, -8.9) |
| p-value |  | 0.002 | <0.001 |
| Change from baseline at day 28 | -5.8 (2.8) | -23.4 (2.9) | -27.2 (2.9) |
| Difference vs placebo (95% CI) |  | -17.6 (-25.8, -9.3) | - 21.4 (-29.6, -13.2) |
| p-value |  | <0.001 | <0.001 |
| **Percentage of time with glucose level ≥70 to <180 mg/dL per day** |  |  |  |
| Baseline | 54.4 (5.0)^†^ | 52.0 (4.3) | 55.0 (6.2) |
| Change from baseline at day 1 | 4.0 (1.9) | 12.7 (2.0) | 17.5 (2.0) |
| Difference vs placebo (95% CI) |  | 8.7 (3.2, 14.3) | 13.5 (8.1, 19.0) |
| p-value |  | 0.003 | <0.001 |
| Change from baseline at day 28 | 5.7 (2.8) | 23.2 (2.9) | 27.3 (2.9) |
| Difference vs placebo (95% CI) |  | 17.6 (9.3, 25.8) | 21.6 (13.5, 29.8) |
| p-value |  | <0.001 | <0.001 |
| **Percentage of time with glucose level <70 mg/dL per day** |  |  |  |
| Baseline | 0.0 (0.0) | 0.2 (0.2) | 0.0 (0.0) |
| Change from baseline at day 1 | -0.2 (0.3) | 0.2 (0.3) | 0.4 (0.3) |
| Difference vs placebo (95% CI) |  | 0.4 (-0.4, 1.3) | 0.6 (-0.2, 1.4) |
| p-value |  | 0.327 | 0.141 |
| Change from baseline at day 28 | 0.1 (0.2) | 0.2 (0.2) | -0.1 (0.2) |
| Difference vs placebo (95% CI) |  | 0.1 (-0.4, 0.7) | -0.3 (-0.8, 0.3) |
| p-value |  | 0.696 | 0.355 |

Baseline data are mean (standard error [SE]), change from baseline data are adjusted mean (SE) based on analysis of covariance (ANCOVA) in the full analysis set. *47.9 (5.3) for day 1 analysis (n=21). ^†^52.1 (5.3) for day 1 analysis (n=21).

**Supplementary Table 3. Changes in blood pressure, pulse rate and weight.**

|  | **Placebo  (n=21)** | **Empagliflozin  10 mg  (n=20)** | **Empagliflozin 25 mg**  **(n=19)** |
| --- | --- | --- | --- |
| **Systolic blood pressure, mmHg** |  |  |  |
| Baseline (day 1) | 119.8 (11.5) | 119.1 (15.9) | 124.0 (11.6) |
| Change from baseline at day 2 | 1.1 (5.8) | -0.4 (7.1) | -2.2 (6.2) |
| Change from baseline at day 29 | 1.3 (9.7) | -4.9 (8.9) | -5.9 (9.6) |
| **Diastolic blood pressure, mmHg** |  |  |  |
| Baseline (day 1) | 71.8 (7.7) | 70.7 (10.7) | 74.7 (8.0) |
| Change from baseline at day 2 | 0.3 (5.0) | 1.0 (4.6) | -1.4 (5.8) |
| Change from baseline at day 29 | 0.7 (6.6) | -1.3 (3.8) | -5.4 (4.4) |
| **Pulse rate (bpm)** |  |  |  |
| Baseline (day 1) | 65.3 (9.9) | 65.3 (8.7) | 64.6 (7.8) |
| Change from baseline at day 2 | 1.4 (5.4) | 4.7 (3.7) | 4.5 (6.5) |
| Change from baseline at day 29 | -1.8 (4.3) | 0.2 (4.8) | -1.7 (5.8) |
| **Weight (kg)** |  |  |  |
| Baseline (day 1) | 67.7 (10.0) | 63.5 (10.6) | 65.9 (12.1) |
| Change from baseline at day 29 | -0.9 (1.0) | -1.7 (0.6) | -2.1 (0.8) |

Data are mean (SD) in patients treated with ≥1 dose of study drug.

**Supplementary Table 4. Laboratory measurements.**

|  | **Placebo** | | **Empagliflozin 10 mg** | | **Empagliflozin 25 mg** | |
| --- | --- | --- | --- | --- | --- | --- |
|  | **Baseline** | **Change from baseline*** | **Baseline** | **Change from baseline*** | **Baseline** | **Change from baseline*** |
| Haematocrit (%)^†^ | 42.3 (4.1) | -2.3 (2.6) | 43.6 (4.2) | -1.0 (2.6) | 43.3 (2.8) | -0.7 (2.7) |
| Uric acid (μmol/l)^†^ | 304 (147) | -4 (43) | 265 (155) | -21 (63) | 268 (84) | -62 (71) |
| eGFR (ml/min/1.73m^2^) (Japanese estimation equation) | 82.6 (12.8) | -2.3 (7.3) | 76.5 (11.1) | -4.7 (4.6) | 80.7 (9.3) | -4.9 (5.8) |
| Electrolytes (mmol/l)^†^ |  |  |  |  |  |  |
| Sodium | 140 (1) | 0 (1) | 139 (2) | 0 (1) | 139 (1) | 0 (1) |
| Potassium | 3.9 (0.2) | 0.0 (0.3) | 4.1 (0.2) | -0.1 (0.2) | 4.0 (0.3) | -0.1 (0.2) |
| Calcium | 2.3 (0.1) | -0.1 (0.1) | 2.3 (0.1) | -0.1 (0.1) | 2.3 (0.1) | -0.1 (0.1) |
| Magnesium | 1.0 (0.1) | 0.0 (0.1) | 1.0 (0.1) | 0.1 (0.0) | 1.0 (0.1) | 0.1 (0.1) |
| Phosphate | 1.2 (0.1) | 0.0 (0.1) | 1.2 (0.1) | 0.0 (0.1) | 1.2 (0.1) | 0.0 (0.1) |

Data are mean (SD) in patients treated with ≥1 dose of study drug. *****Change from baseline at last value on treatment for hematocrit, uric acid and electrolytes; change from baseline at day 28 for eGFR. ^†^Normalized to a standard reference range.
eGFR, estimated glomerular filtration rate.
